# Prospective Analysis of Arteriovenous Fistula Performance in the Context of Competing Risks

**DOI:** 10.34067/KID.0000000650

**Published:** 2024-11-19

**Authors:** Anukul Ghimire, Anita M. Lloyd, Susan Szigety, Jose Luis Merino, Karim Alibhai, Gerrit Winkelaar, Robert R. Quinn, Marcello Tonelli

**Affiliations:** 1 Department of Medicine, University of Calgary, Calgary, Alberta, Canada; 2 Department of Medicine, University of Alberta, Edmonton, Alberta, Canada; 3 Department of Nephrology, Hospital Universitario del Henares, Coslada, Spain

**Keywords:** arteriovenous fistula, dialysis access, hemodialysis, vascular access

## Abstract

**Key Points:**

- Among 257 newly created arteriovenous fistulas, primary nonfunction occurred in 49%, and only 55% were ultimately used for dialysis.
- Loss of arteriovenous fistula patency was lower when competing risks were accounted for compared with conventional Kaplan–Meier analysis.
- We present icon-array plots that summarize our data and may be used a decision aid for patients in the future.

**Background:**

Many patients with newly created arteriovenous fistulas (AVFs) may die before the AVF is needed for hemodialysis. However, formal competing risks (CRs) frameworks are rarely used to report AVF patency, which may lead to biased estimates. We sought to identify the proportion of newly created AVF experiencing primary nonfunction and describe long-term patency using a CR framework.

**Methods:**

We conducted a prospective observational study in 257 adults with newly created AVF in Alberta, Canada. The primary outcome was primary nonfunction. Secondary outcomes included loss of primary patency, loss of assisted primary patency, and loss of secondary functional patency. Results were presented using icon-array plots to form the basis for future decision aids.

**Results:**

Participants were 63.0% male, with mean age 62.3 years and median follow-up 18.5 months (range, 0.02–180 months). Of 257 participants, 50 could not be assessed for function or primary nonfunction, usually because of death. Of the remaining 207, 102 (49.3%) had primary nonfunction, and function was ultimately established for 142 (68.6%). Thus, only 142 of the 257 participants (55.3%) ultimately used the AVF for hemodialysis. High rates of CRs led to biased results from Kaplan–Meier analyses of lost patency. When accounting for CRs, loss of primary patency among AVFs with established function was 36.6%, 65.5%, and 66.2%, at 1, 3, and 5 years, respectively.

**Conclusions:**

Only 55% of fistulas were ultimately used for hemodialysis when accounting for CRs and primary nonfunction. These results and the icon-array plots may inform discussions surrounding vascular access options for patients.

## Visual Abstract

**Figure:**
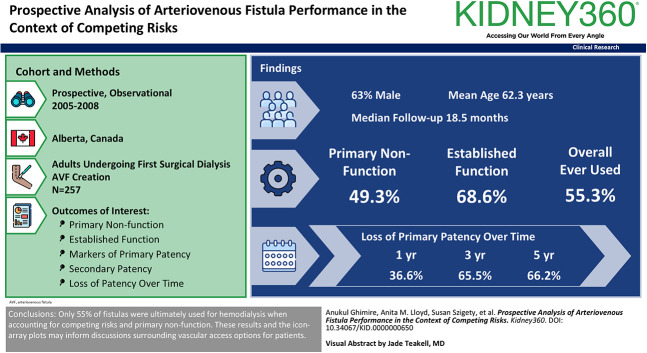

## Introduction

Native vessel arteriovenous fistulas (AVFs) are associated with better patency, lower infection rates, and lower costs compared with other forms of vascular access for hemodialysis.^1–6^ On the basis of these observational data, national guidelines previously recommended AVF as the preferred form of vascular access,^7–9^ and large-scale quality improvement schemes, such as the Fistula First Breakthrough initiative, were established to increase the proportion of patients receiving hemodialysis through AVF.^10^ Once created, AVF usually require 8–12 weeks to mature, during which time the blood flow rates through the fistula increase as the native veins dilate. Ideally, AVF should be created well before dialysis treatment is required to allow adequate maturation. However, approximately 25% of AVF may never mature sufficiently to permit hemodialysis treatment, which is termed as primary nonfunction.^11–13^

Contemporary recommendations emphasize that the choice of vascular access should be individualized on the basis of patient-specific factors, such as a patient's ability to tolerate needling pain, vascular anatomy, availability of professional workforce to create and monitor the vascular access, and likelihood that the AVF will ever be used.^2^ For example, elderly patients may be more likely to have primary nonfunction and/or die before their fistula is usable.^14,15^ Detailed assessments of primary nonfunction, the proportion of AVFs that are ultimately usable for hemodialysis, and loss of patency over the long term may inform this discussion, as would information on how these outcomes vary by patient characteristics.

Previous work has shown that up to 60% of elderly patients who undergo AVF creation may never use the fistula because of either nonmaturation or death occurring before dialysis initiation.^11,15–17^ These studies, however, have not consistently used a competing risk (CR) framework to account for events such as death and modality change when reporting rates of primary function and long-term outcomes, which may have led to biased estimates. Furthermore, most studies characterizing AVF patency and nonfunction have a retrospective design or are limited by short follow-up time.

We designed this prospective study to determine the proportion of participants with a newly constructed AVF with primary nonfunction; the proportion in which AVF is ultimately usable for hemodialysis; and the loss of fistula patency over time while accounting for CRs, such as death. An important secondary goal was to create icon-array plots^18^ to summarize the key results. Visual aids have shown to improve patient understanding of probabilistic information, and icon-array plots are a type of visual aid that can effectively convey information to a wide range of patient groups.^19,20^ Use of icon-array plots may enhance discussions between patients and providers about the most appropriate vascular access type.

## Methods

### Study Design and Inclusion Criteria

This was a prospective observational study in the Alberta Kidney Care-North program in Alberta, Canada. The inclusion criteria were adults (aged 18 years and older) undergoing access surgery for creation of an AVF between July 2005 and November 2008. There were five surgeons involved in the creation of AVFs. Participants undergoing creation of a polytetrafluoroethylene graft, those undergoing a revision of either fistula or graft, or those without informed consent were excluded (Supplemental Figure 1). Study visits continued until access failure/abandonment/ligation/removal, death, out-migration, refusal to use the access, recovery of function, receipt of a kidney transplant, commencement of peritoneal dialysis (PD), withdrawal of consent, or 15 years after AVF creation, whichever occurred earliest. This analysis included only the first AVF per participant during the study period.

### Data Collection

Consenting participants underwent a structured interview and chart review at baseline to collect detailed data on demographic characteristics and medical history. Follow-up visits were performed by the study assistant at 3 months, 6 months, 1 year, and then yearly after access surgery. A standardized data collection form and corresponding database for baseline and follow-up visits were created before study commencement. Laboratory data and vital status were collected by chart review or linkage to data from the provincial health ministry. The University of Alberta Health Research Ethics Board approved the study (Pro00001323).

### Outcome Measures

The primary outcome for this study was primary nonfunction. Secondary outcomes included established function and markers of primary patency, assisted primary patency, and secondary patency (Supplemental Figure 2). Death, out-migration from the province, withdrawal of consent, recovery of kidney function, switch to PD, and kidney transplantation were treated as competing events that preclude the occurrence of patency loss. Given the relatively large number of competing events and the potential for these events to cause biased estimates of patency, we elected to present results in terms of lost patency using a CR framework.^21^ Therefore, the secondary outcomes were expressed as loss of primary patency, loss of assisted primary patency, and loss of secondary patency.

To differentiate between AVFs that were patent but had not yet been assessed for established function (*e.g*., in a participant who had not yet commenced kidney replacement), we also considered loss of primary functional patency, loss of assisted primary functional patency, and loss of secondary functional patency. As an example, primary patency starts at the time of AVF creation, whereas primary functional patency starts on the date of the third consecutive dialysis run with average blood flow ≥300 ml/min. Detailed definitions for all outcomes are presented in Table 1; patency outcomes are depicted in Supplemental Figure 2.

**Table 1:** Definitions

Outcome	Definition
Primary patency	Primary patency starts at the date of access creation Primary patency ends when any of the following occur: • any intervention designed to maintain/re-establish patency; • any of access thrombosis, no thrill or bruit, or occlusion; or • access failure/abandonment/ligation/removal Interventions designed to maintain or re-establish patency include angioplasty, declot, surgical revision Time to the end of primary patency is censored for participants who die, move out of province, recover function, receive a kidney transplant, start chronic PD, withdraw consent, or reach the end of follow-up
Assisted primary patency	Assisted primary patency starts on the date of access creation Assisted primary patency ends when any of the following occur: • any of access thrombosis, no thrill or bruit, or occlusion; or • access failure/abandonment/ligation/removal Interventions to maintain patency are ignored Time to the end of assisted primary patency is censored for participants who die, move out of province, recover function, receive a kidney transplant, start chronic PD, withdraw consent, or reach the end of follow-up
Secondary patency	Secondary patency starts on the date of access creation Secondary patency ends at: • access failure/abandonment/ligated/removal Interventions to re-establish patency or any instances of access thrombosis, no thrill or bruit, or occlusion are ignored Time to the end of secondary patency is censored for participants who die, move out of province, recover function, receive a kidney transplant, start chronic PD, withdraw consent, or reach the end of follow-up
Established function	Established function occurs when the access has been used for least three consecutive dialysis sessions with average blood flow ≥300 ml/min If function is not established before study exit because of death, move out of province, recover function, receive a kidney transplant, start chronic PD, withdraw consent, or reach the end of follow-up, then function is considered as “not assessed”
Primary nonfunction	Primary nonfunction occurs when any of the following occur: • AVF undergoes intervention to maintain/establish patency (angioplasty, declot, surgical revision) at any point in time before reaching established function; • participant refuses to use AVF before AVF function is assessed; or • access failure/abandonment/removal before AVF function is assessed
Primary functional patency	Primary functional patency starts on the date of established function (*i.e*., the date of the third consecutive dialysis run with average blood flow ≥300 ml/min) Primary functional patency ends when any of the following occur: • any intervention designed to maintain/re-establish patency; • any of access thrombosis, no thrill or bruit, or occlusion; or • access failure/abandonment/ligated/removal Interventions designed to maintain or re-establish patency include angioplasty, declot, or surgical revision Time to the end of primary functional patency is censored for participants who die, move out of province, recover function, receive a kidney transplant, start chronic PD, withdraw consent, or reach the end of follow-up Primary functional patency is assessed only for participants with established function
Assisted primary functional patency	Assisted primary functional patency starts on the date of established function (*i.e*., the date of the third consecutive dialysis run with average blood flow ≥300 ml/min) Assisted primary functional patency ends when any of the following occur: • any of access thrombosis, no thrill or bruit, or occlusion; or • access failure/abandonment/ligation/removal Interventions to maintain patency are ignored Time to the end of assisted primary functional patency is censored for participants who die, move out of province, recover function, receive a kidney transplant, start chronic PD, withdraw consent, or reach the end of follow-up Assisted primary functional patency is assessed only for participants with established function
Secondary functional patency	Secondary functional patency starts at the date of established function (*i.e*., the date of the third consecutive dialysis run with average blood flow ≥300 ml/min) Secondary functional patency ends at: • access failure/abandonment/ligation/removal Notes Interventions to re-establish patency or any instances of access thrombosis, no thrill or bruit, or occlusion are ignored Time to the end of secondary functional patency is censored for participants who die, move out of province, recover function, receive a kidney transplant, start chronic PD, withdraw consent, or reach the end of follow-up Secondary functional patency is assessed only for participants with established function

AVF, arteriovenous fistula; PD, peritoneal dialysis.

### Statistical Analyses

We reported baseline descriptive statistics as counts and percentages, means and SDs, or medians and interquartile ranges (IQRs), as appropriate. We calculated the proportion of participants who experienced established function and primary nonfunction, considering the entire follow-up period. Next, we calculated patency at 1, 3, and 5 years after access creation using Kaplan–Meier (KM) methods. We censored for the following reasons: participant death, relocation, refusal to use the access, recovery of kidney function, modality switch (to PD or kidney transplant), withdrawal of consent, or end of follow-up. Second, because KM methods may yield biased estimates in the presence of CRs, we converted the proportion experiencing patency to the proportion experiencing failure (*i.e*., the one- KM estimate) and compared these results with analyses using a CR framework.^22^ While end of follow-up remained a censoring event, the remaining censoring reasons were treated as CRs. Missing values were singly imputed with the most frequent categorical or median continuous value. We conducted statistical analyses using Stata/MP 18.0 software (www.stata.com).

### Visual Presentation

We summarized the key results using an icon-array plot.^23^ Icon-array plots are a type of visual representation used to display the frequency or proportion of categorical variables. Icon-array plots can be used as the basis for a decision aid because their interpretation is often intuitive, and they can effectively convey statistical information, even to people with limited numeracy.^18^

## Results

### Study Participants

We approached 316 adults undergoing vascular access surgery, and 257 were included (Supplemental Figure 1). Baseline characteristics are presented in Table 2. At the time of cohort entry, participants were predominantly White (77.4%); 63.0% were male with a mean age of 62.3 years. Approximately half of participants were in the two lowest income quintiles, and 17.1% lived in a rural area. The most common comorbidities included hypertension (89.5%), diabetes (56.4%), connective tissue disease (31.1%), and ischemic heart disease (27.6%).

**Table 2:** Baseline characteristics of participants

Characteristic	*N*=257
Age, yr, mean (SD)	62.3 (15.8)
Female	95 (37.0)
**Ethnicity**	
Asian	6 (2.3)
Black	4 (1.6)
Indian subcontinent, Middle East/Arabian, Pacific Islander	13 (5.1)
Indigenous	32 (12.5)
Unknown	3 (1.2)
White	199 (77.4)
**Education**	
Unknown	142 (55.3)
High school graduate or less	88 (34.2)
Post secondary	27 (10.5)
**Employment status**	
Retired	130 (50.6)
Disabled	44 (17.1)
Employed or student (full or part-time)	32 (12.5)
Unknown	28 (10.9)
Unemployed	23 (8.9)
**Socioeconomic status**	
1 (lowest income quintile)	82 (31.9)
2	47 (18.3)
3	52 (20.2)
4	44 (17.1)
5 (highest income quintile)	32 (12.5)
Rural	44 (17.1)
SBP/DBP, mm Hg, mean (SD)	134 (22)/74 (15)
BMI, kg/m^2^, median (IQR)	27.9 (24.3–32.9)
**Smoking status**	
Former smoker	112 (43.6)
Nonsmoker	81 (31.5)
Active smoker	51 (19.8)
Unknown	13 (5.1)
First permanent access ever	117 (45.5)
**Comorbidities**	
Hypertension	230 (89.5)
Diabetes	145 (56.4)
Connective tissue disease	80 (31.1)
Ischemic heart disease	71 (27.6)
Pulmonary disease	66 (25.7)
Chronic pain	59 (23.0)
Congestive heart failure	48 (18.7)
Peripheral vascular disease	44 (17.1)
Depression	44 (17.1)
Hypothyroidism	40 (15.6)
Malignancy	36 (14.0)
Peptic ulcer disease	32 (12.5)
Atrial fibrillation	27 (10.5)
Alcohol misuse	26 (10.1)
Cerebrovascular disease	25 (9.7)
Liver problem	18 (7.0)
**Cause of kidney failure (>1 cause is possible)**	
Diabetes	110 (42.8)
GN	63 (24.5)
Hypertension/kidney vascular disease	38 (14.8)
Interstitial nephritis/nephropathy	15 (5.8)
Unknown etiology	10 (3.9)
Polycystic kidney disease	8 (3.1)
Other	7 (2.7)
Obstructive uropathy	6 (2.3)
Lupus	5 (1.9)
Drug toxicity	4 (1.6)
Pyelonephritis	3 (1.2)
**Dialysis status at the time of AVF creation**	
On hemodialysis with CVC	134 (52.1)
Nondialysis CKD	109 (42.4)
Using functioning transplant	6 (2.3)
On PD	5 (1.9)
On hemodialysis with previous fistula/graft	3 (1.2)
**Laboratory results, median (IQR)**	
eGFR, ml/min per 1.73 m^2^^a^	12.2 (10.2–15.1)
Serum creatinine, *μ*mol/L^a^	389.4 (323.8–478.4)
Serum creatinine, *μ*mol/L^b^	645 (473–829)
Calcium, mmol/L	2.2 (2.1–2.3)
CO_2_, mmol/L	23 (20–25)
Hemoglobin, g/L	105 (98–115)
Parathyroid hormone, pmol/L	22.3 (13.3–32.6)
Phosphate, mmol/L	1.7 (1.5–1.9)
Predialysis urea, mmol/L	23.5 (17.8–29.8)

*N* (%) unless otherwise specified. Baseline characteristics were assessed at study entry. AVF, arteriovenous fistula; BMI, body mass index; CO_2_, carbon dioxide; CVC, central venous catheter; DBP, diastolic BP; IQR, interquartile range; PD, peritoneal dialysis; SBP, systolic BP.

a eGFR and serum creatinine for the 115 participants who had not commenced dialysis or had a functioning kidney transplant at the time of cohort entry.

b Serum creatinine values for participants receiving hemodialysis were obtained immediately before the hemodialysis session closest to study entry.

Median follow-up was 18.5 months (IQR, 5.5–45.6). At the time of access creation, 137 (53.3%) were receiving maintenance hemodialysis using a central venous catheter (CVC) (*n*=134) or a previous fistula/graft (*n*=3). Among those not receiving hemodialysis (*n*=120, 46.7%), most had not yet initiated kidney replacement (*n*=109), six had a functional but failing kidney transplant, and five were receiving PD (Table 2). One hundred seventeen of the 257 AVFs were the first permanent vascular access for that participant.

### Primary Nonfunction and Established Function

Primary nonfunction and established function could be evaluated in 207 of 257 AVFs; these outcomes could not be assessed in 44 of 257 (17.1%) because of death (*n*=40) or change in the kidney replacement modality (*n*=4). Among the 40 participants who died before the newly created AVF was used for hemodialysis, 18 had been receiving treatment through hemodialysis (*n*=16 using a CVC, *n*=1 using a previous fistula or graft) or PD (*n*=1), while 22 had not yet initiated kidney replacement; the median time to death was 0.5 years (IQR, 2 months–1.3 years). Among the four participants who changed modality before the AVF was used for hemodialysis, two were already being treated with hemodialysis using CVC and two had not yet initiated kidney replacement. An additional six participants either withdrew consent or moved away before AVF function could be evaluated. Thus, 50 participants (death *n*=40, modality switch *n*=4, and withdrew consent or moved away *n*=6) were not evaluated for primary nonfunction or established function.

Among the 207 AVFs that were evaluated, primary nonfunction occurred in 102 (49.3%). Of these 102 AVFs, function was established after an intervention in 37. An additional 105 of 207 AVFs had primary function, resulting in a total of 142 of 207 (68.6%) with established function. Among the 105 participants with primary function, 65 (62%) were on dialysis at baseline (*n*=64 CVC; *n*=1 previous fistula/graft). The remaining participants were on PD (*n*=1/105), using a previous transplant (*n*=3/105), and not on dialysis yet (36/105).

The remainder of the 207 who were evaluated (*n*=65) never used their access for dialysis and ultimately experienced access failure/abandonment/removal. These participants were evaluated as not having established function and as having primary nonfunction.

**Figure 1:**
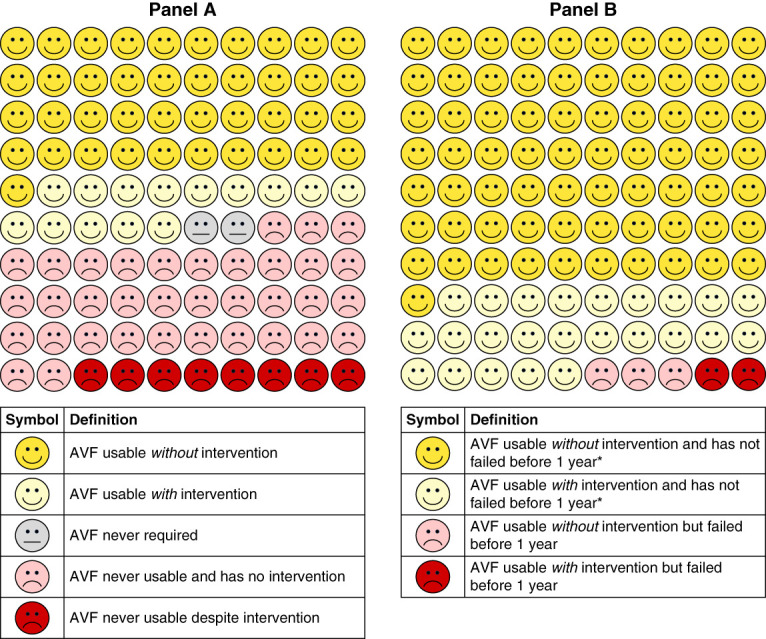
**Icon-array plots showing clinically relevant outcomes.** (A) Initial outcomes for all participants (*n*=257). (B) Subsequent outcomes for those with established AVF function (*n*=142). (A) AVF never required for hemodialysis because of participants switching or choosing another modality to initiate KRT (*i.e*., kidney transplant, chronic PD, or conservative care). (B) Timing is based on time elapsed since the date of established function. *This category includes fistulas that are functional at 1 year and those that were censored before 1 year. Censoring reasons include death, modality switch (kidney transplant, chronic PD, recovery of function), or moving away. AVF arteriovenous fistula; PD, peritoneal dialysis.

**Figure 2:**
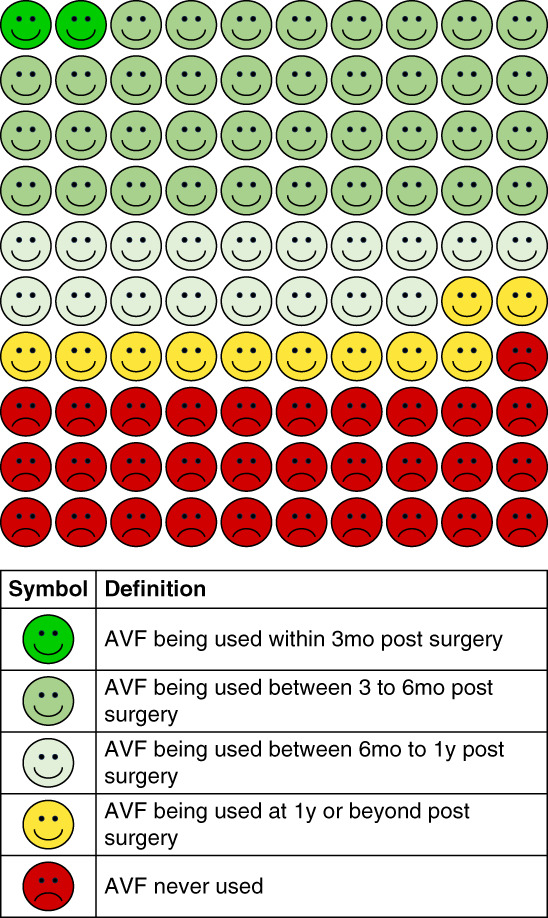
**Icon-array plots showing proportion of newly created AVFs that were usable within a certain time after access creation.** Analysis included all AVFs where function could be assessed (*n*=207).

Among the 207 where function could be assessed, 4 (1.9%) were used for dialysis within 3 months of creation and 22 (10.6%) were not used within the first year. Among the 142 AVFs with established function, 4 (2.8%) had established function established within 3 months, 79 (55.6%) had established function between 3 and 6 months, 37 (26.1%) had established function between 6 months and 1 year, and 22 (15.5%) had established function after 1 year of access creation. The median time to established function from access creation was 0.46 years (IQR, 0.35–0.75) for the 142 AVFs with established function. When considering all 257 AVFs, 142 (55.3%) were ultimately used for dialysis. The results from these analyses were presented using icon-array plots (Figures 1 and 2). In Figure 1, the “AVF never required” icon represents the 4 of 257 participants switching or choosing another modality to initiate KRT (*i.e*., kidney transplant, chronic PD, or conservative care). The “AVF never usable and has no intervention” icon represents 52 patients with access failure, 33 who died, four who moved, and one who withdrew consent (90/257, 35%). The “AVF never usable despite intervention” icon represents 13 patients with access failure, seven who died, and one who withdrew consent (21/257, 8%).

Supplemental Figure 3 shows how the likelihood of established function and primary nonfunction varies by baseline patient characteristics. The likelihood of primary nonfunction tended to be greater in older patients, female patients, patients with lower arm AVF, and those having their first-ever AVF creation (all *P* < 0.05). Female participants and those with lower arm AVFs were less likely to ultimately achieve established function (both *P* < 0.05).

### Primary Patency

Among the 142 AVFs with established function, 1-, 3-, and 5-year primary patency as calculated using the KM method was 62.4%, 24.1%, and 22.4%, respectively. However, during follow-up, primary patency was censored for 45 (31.7%) AVFs, mostly because of death (*n*=26) and kidney transplantation (*n*=16, Supplemental Table 3). Primary patency was lost because of angioplasty or surgery to maintain patency for 83 (58.5%), thrombosis or occlusion for 12 (8.5%), and access abandonment for 2 (1.4%).

### Loss of Primary Patency

**Figure 3:**
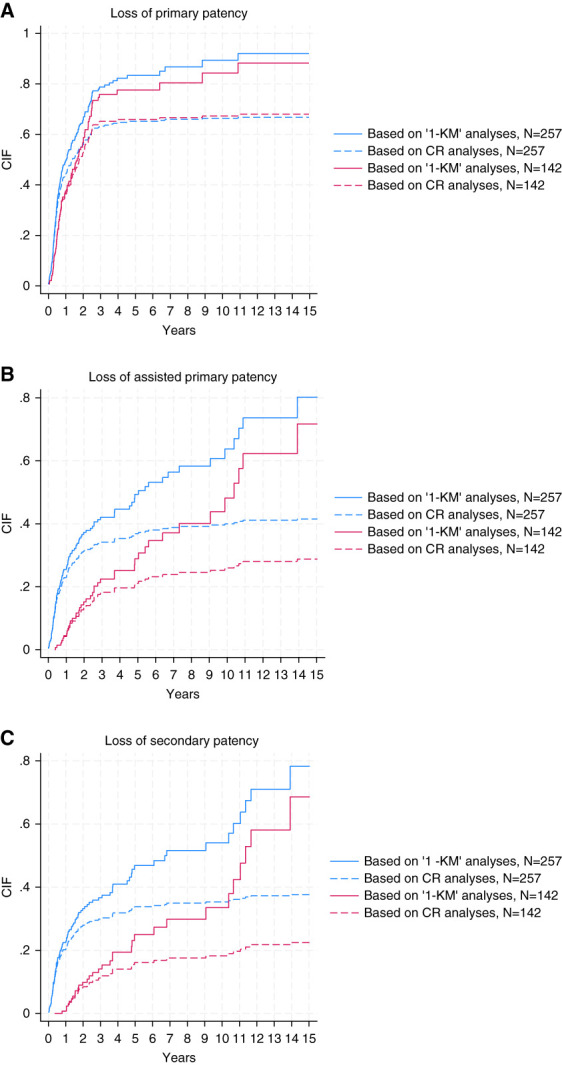
**Plots showing cumulative incidence of outcomes of interest on the basis of both KM and CR analyses.** (A) Loss of primary patency (B) Loss of assisted primary patency (C) Loss of secondary patency. *N*=142: including only AVF with established function. *N*=257: analyses including all newly created AVFs. CIF, cumulative incidence function; CR, competing risk; KM, Kaplan–Meier.

Outcomes for the CR analyses are expressed as loss of patency. After accounting for CRs, the proportion with loss of primary patency among the 142 AVFs with established function was 36.6% at 1 year (95% confidence interval [CI], 28.8% to 44.5%), 65.5% at 3 years (95% CI, 57.1% to 72.7%), and 66.2% at 5 years (95% CI, 57.8% to 73.3%). When considering all 257 AVFs and accounting for CRs, the proportion with loss of primary patency at 1, 3, and 5 years was 44.4% (95% CI, 38.2% to 50.3%), 63.4% (95% CI, 57.2% to 69.0%), and 65.4% (95% CI, 59.2% to 70.8%), respectively. These proportions are lower than the results from the comparable KM results (Figure 3 and Table 3) and demonstrate the bias introduced by neglecting the CRs. When considering only AVFs that were the first permanent access for participants (*i.e*., not including AVFs that were the second or subsequently created AVF), the results for loss of primary patency were similar (Supplemental Table 1).

**Table 3:** Patency outcomes

Outcome	1-yr CR	1-yr KM	3-yr CR	3-yr KM	5-yr CR	5-yr KM
**Including only AVF with established function (*N*=142)**						
Loss of primary patency	36.6 (28.8 to 44.5)	37.6 (30.1 to 46.2)	65.5 (57.1 to 72.7)	75.9 (67.4 to 83.6)	66.2 (57.8 to 73.3)	77.6 (68.9 to 85.3)
Loss of assisted primary patency	4.2 (1.7 to 8.5)	4.5 (2.0 to 9.7)	18.3 (12.5 to 25.1)	22.5 (15.8 to 31.4)	21.1 (14.8 to 28.2)	28.9 (20.6 to 39.5)
Loss of secondary patency	0.7 (0.1 to 3.5)	0.8 (0.1 to 5.2)	11.3 (6.7 to 17.1)	14.1 (8.8 to 22.1)	16.2 (10.7 to 22.7)	25.0 (16.9 to 36.1)
**Including all newly created AVFs (*N*=257)**						
Loss of primary patency	44.4 (38.2 to 50.3)	49.4 (43.1 to 56.2)	63.4 (57.2 to 69.0)	78.7 (72.3 to 84.6)	65.4 (59.2 to 70.8)	83.4 (76.9 to 88.9)
Loss of assisted primary patency	23.0 (18.0 to 28.3)	25.5 (20.3 to 31.7)	34.2 (28.5 to 40.1)	42.0 (35.4 to 49.3)	37.0 (31.1 to 42.8)	49.3 (41.7 to 57.6)
Loss of secondary patency	20.2 (15.6 to 25.3)	22.4 (17.6 to 28.4)	30.0 (24.5 to 35.6)	36.6 (30.3 to 43.8)	33.9 (28.1 to 39.7)	46.9 (39.1 to 55.4)

Loss of patency is presented as % (with corresponding 95% confidence interval).

For competing risk analyses, the loss of patency is based on the cumulative incidence. Death, moving out of province, recovery of function, receiving a kidney transplant, starting chronic peritoneal dialysis, and withdrawal of consent are treated as competing risks. Participants are censored at the end of follow-up.

For Kaplan–Meier analyses, the loss of patency is based on the one-Kaplan–Meier estimator. Participants are censored for death, moving out of province, recovery of function, receiving a kidney transplant, starting chronic peritoneal dialysis, withdrawal consent, or reaching the end of follow-up.

Time of patency for the primary, assisted primary, and secondary patency measures begins at the time of arteriovenous fistula creation. AVF, arteriovenous fistula; CR, competing risk; KM, Kaplan–Meier.

### Loss of Secondary Patency

Among the 142 AVFs with established function, when CRs were accounted for, the proportion with loss of secondary patency at 1, 3, and 5 years was 0.7% (95% CI, 0.1% to 3.5%), 11.3% (95% CI, 6.7% to 17.1%), and 16.2% (95% CI, 10.7% to 22.7%), respectively. When considering all 257 AVFs and accounting for CRs, the proportion with loss of secondary patency at 1, 3, and 5 years were again lower than the results from KM estimates: 20.2% (95% CI, 15.6% to 25.3%), 30.0% (95% CI, 24.5% to 35.6%), and 33.9% (95% CI, 28.1% to 39.7%), respectively (Figure 3 and Table 3). When considering only AVFs that were the first permanent access for participants, the results for loss of secondary patency were similar (Supplemental Table 1).

The results for primary and secondary functional patency were similar to those from these previous analyses and are presented in Supplemental Table 2.

## Discussion

This prospective cohort study of 257 participants undergoing AVF creation for hemodialysis treatment had four key findings. First, primary nonfunction occurred in 102 of 207 AVFs (49%), in whom this outcome could be assessed and was more common in the elderly, female patients, patients with a lower arm AVF, and those having their first-ever AVF creation. The remaining 105 AVFs had primary function and so did not require any intervention before use. Second, function was ultimately established in 142 of 207 AVFs (69%), but the remainder (*n*=65) could not be used for hemodialysis treatment. Third, 17.1% (44/257) of all participants died or changed their planned kidney replacement modality and, as a result, never needed their AVF for dialysis. Therefore, of 257 participants undergoing AVF creation, only 55% ultimately used that AVF for hemodialysis. Fourth, failing to account for CRs leads to biased results that overestimate the likelihood of lost patency among AVFs with established function. However, even after accounting for CRs, 20.2%, 30.0%, and 33.9% of the 257 patients undergoing AVF creation had fistulas that were not being used for hemodialysis at 1, 3, and 5 years, respectively.

Our findings are similar to those from other studies suggesting that approximately 35% of created AVFs are never used^24,25^ and that the loss of primary patency at 3 years is approximately 70%.^26^ These previous reports tend to be retrospective and typically ignore competing events, such as death and modality switches, when calculating AVF performance.^25–27^ Our results confirm that the loss of AVF patency is overestimated when follow-up is censored at the time of competing events, suggesting that future studies of hemodialysis vascular access should consistently use a CR framework to report results.^21^ On the other hand, analyses that exclude patients in whom the AVF is never used will tend to overestimate the clinical value of AVF creation. Previous studies using CRs to highlight AVF patency are limited in number and have not shown differences in assisted patency using CR versus conventional analysis.^28,29^ These studies have typically included only AVF undergoing procedures to establish or maintain patency (rather than newly created AVF) and do not consistently include CRs besides death (*e.g*., modality change).

There are other notable gaps in available studies describing the natural history of AVF. First, prior studies often excluded patients with primary nonfunction, which may have led to overestimation of the clinical utility of creating AVF.^30–33^ Second, the follow-up periods in these studies are typically limited to only a few years, with longer term outcomes being mainly reported in retrospective analysis^30,34,35^ rather than prospective cohorts.^25,36^ Third, there is substantial between-cohort heterogeneity in outcomes reported in systematic reviews of AVF performance, with variable inclusion criteria and inconsistent definitions of AVF patency.^13,31,37^ These observations have implications for future studies that seek to inform the choice of vascular access in patients receiving hemodialysis.

Our study addresses these gaps and provides an icon-array plot that may help with shared decision making about the choice of hemodialysis access, including risk of nonmaturation, fistula patency rates, and probability of future interventions.^38^ Icon-array plots enhance comprehension of ratios and lead to better estimates of risk compared with other formats^39^ and have been effective in communicating medical risks in various settings.^40–42^ These plots have been shown to more effectively convey health-related information to the elderly and those with low numeracy skills,^20^ which may represent a large proportion of patients on contemporary dialysis.^43^ Despite this, the use of icon-array plots in dialysis care has been limited to date. Our icon-array plots provide a clinically relevant and simple summary of relatively complex statistical models and may help patients better comprehend the risks of AVF failure and need for interventions to maintain patency. The clinical utility and patient perceptions to our icon-array plots warrants further investigation. Future work may also consider creating icon-array plots to highlight the risks associated with tunneled dialysis catheters and arteriovenous grafts.

It is well known that many patients with advanced CKD die before ever needing dialysis, and predialysis planning in these settings may be wasteful.^44^ Predicting which patients will use the AVF at the time of dialysis initiation is difficult,^45^ but earlier AVF placement is thought to allow for greater time to maturation, thus potentially enhancing the proportion of patients who commence dialysis with a functional AVF. On the other hand, the high proportion of participants who never required their AVF in our study illustrates the potential downside of this approach. These findings have implications for policy in lower income countries where health care resources are scarce and workforce capacity is limited.^46–49^ In high-income countries, with older and more complex dialysis populations,^50^ there may be increased rates of primary nonfunction and death occurring before dialysis initiation.^14^ This may also have implications for performance measures and reimbursement policies. Principle payors, such as the Center for Medicare & Medicaid services, have historically promoted fistula use.^51^ Our results suggest that performance measures detailing vascular access should be reported with consideration of CRs to limit bias. As an example, centers should not be penalized if they serve an older dialysis population (with higher likelihood of both primary nonfunction and CR of death), where promoting higher uptake of fistulas may not be the most cost-effective option.^52^

Our study has important strengths, including its use of internationally recognized definitions for access patency and long-term follow-up of patients treated in a universal health care system. Our comparison of long-term AVF patency using conventional and CR models is pragmatic and highlights the historic limitations in this field. However, our results should be interpreted in the context of their limitations. First, our analysis was based on participants drawn from a single dialysis program in Alberta, with nearly 90% of the participants being White or Indigenous. As such, our findings may not be generalizable to other populations.^53^ Furthermore, the influence of surgical training and practice patterns, which are important considerations for vascular access outcomes,^54^ could not be analyzed given the single center nature of our study. Our study also did not capture data regarding the experience of surgeons or interventionalists. Second, the mean age in our cohort (62.3 years) was typical of the Canadian hemodialysis population, but this means that our overall conclusions may not apply to younger patients or those with fewer comorbidities. Third, the sample size of our cohort was relatively small, although this was mitigated by our prospective collection of a wide range of clinically important variables. Furthermore, we do not report data on the rate of interventions required to maintain AVF functionality, and we did not follow participants after failure of AVF.

In summary, primary nonfunction occurred in nearly half of newly created AVF, and only 55% of all AVFs were ultimately used for hemodialysis treatment, often because of CRs, such as death or modality switches. Our work supports the need to individualize vascular access type for each patient and to consider the potential disadvantages of early AVF placement, including primary nonfunction and CRs. Furthermore, we show that loss of AVF patency is overestimated when CRs are ignored. Future studies of vascular access outcomes should consider using a CR framework to avoid biased results. The icon-array plots that summarize our findings may help to guide discussions between patients and providers about the most appropriate vascular access type. Further work is needed to design and validate decision aids on the basis of these plots and to demonstrate their utility in clinical practice.

## Supplementary Material

## Data Availability

Partial restrictions to the data and/or materials apply. This study is based in part on data provided by Alberta Health and Alberta Health Services. The interpretation and conclusions contained herein are those of the researchers and do not necessarily represent the views of the Government of Alberta or Alberta Health Services. Neither the Government of Alberta nor, Alberta Health or Alberta Health Services express any opinion in relation to this study.

## Supplemental Material

Supplemental Figure 1. Flow diagram.

Supplemental Figure 2. Illustrative depiction of patency outcomes for sample AVF.

Supplemental Figure 3. Plot of baseline characteristics.

Supplemental Table 1. Patency outcomes for AVFs that are first permanent access for participants.

Supplemental Table 2. Other patency outcomes.

Supplemental Table 3. Reasons for censoring, by outcome.
