# Supplementary material for: Prospective Analysis of Arteriovenous Fistula Performance in the Context of Competing Risks
Source: Kidney360. 2024 Nov 19;6(2):272–83. doi: 10.34067/KID.0000000650 (PMC11882251; doi:10.34067/KID.0000000650)
Supplement: Supplementary file 2 [file kidney360-6-272-s002.pdf]

Supplemental Material

Supplemental Figure 1: Flow diagram

Supplemental Figure 2: Illustrative depiction of patency outcomes for sample AVF

Supplemental Figure 3: Plot of baseline characteristics

Supplemental Table 1: Patency outcomes for AVF that are first permanent access for participant

Supplemental Table 2: Other patency outcomes

Supplemental Table 3: Reasons for censoring, by outcome

Supplemental Figure 1: Flow diagram

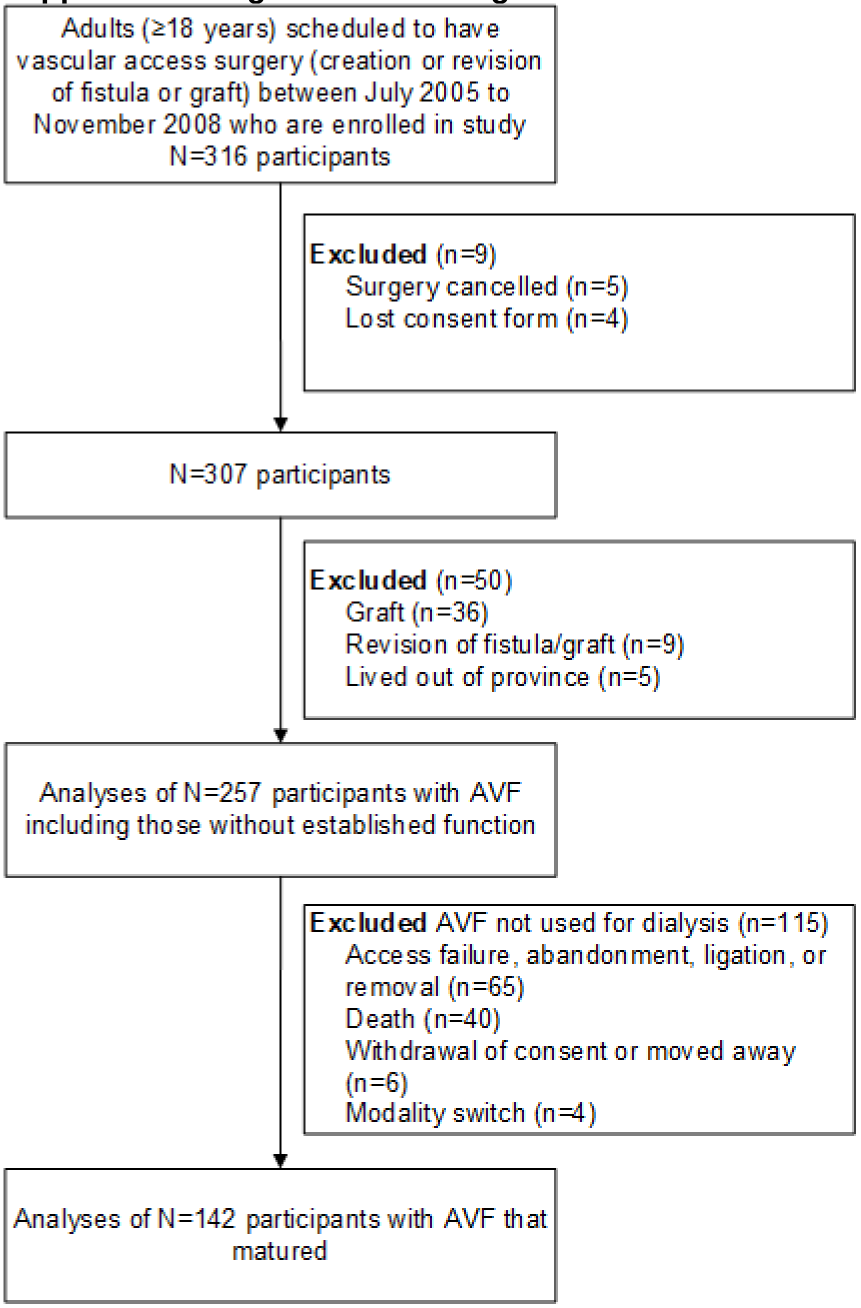

Modality switches include those among participants being treated with hemodialysis and participants with pre-dialysis chronic kidney disease who receive a kidney transplant or commence maintenance PD

AVF arteriovenous fistulas; PD peritoneal dialysis

Supplemental Figure 2: Illustrative depiction of patency outcomes for sample AVF

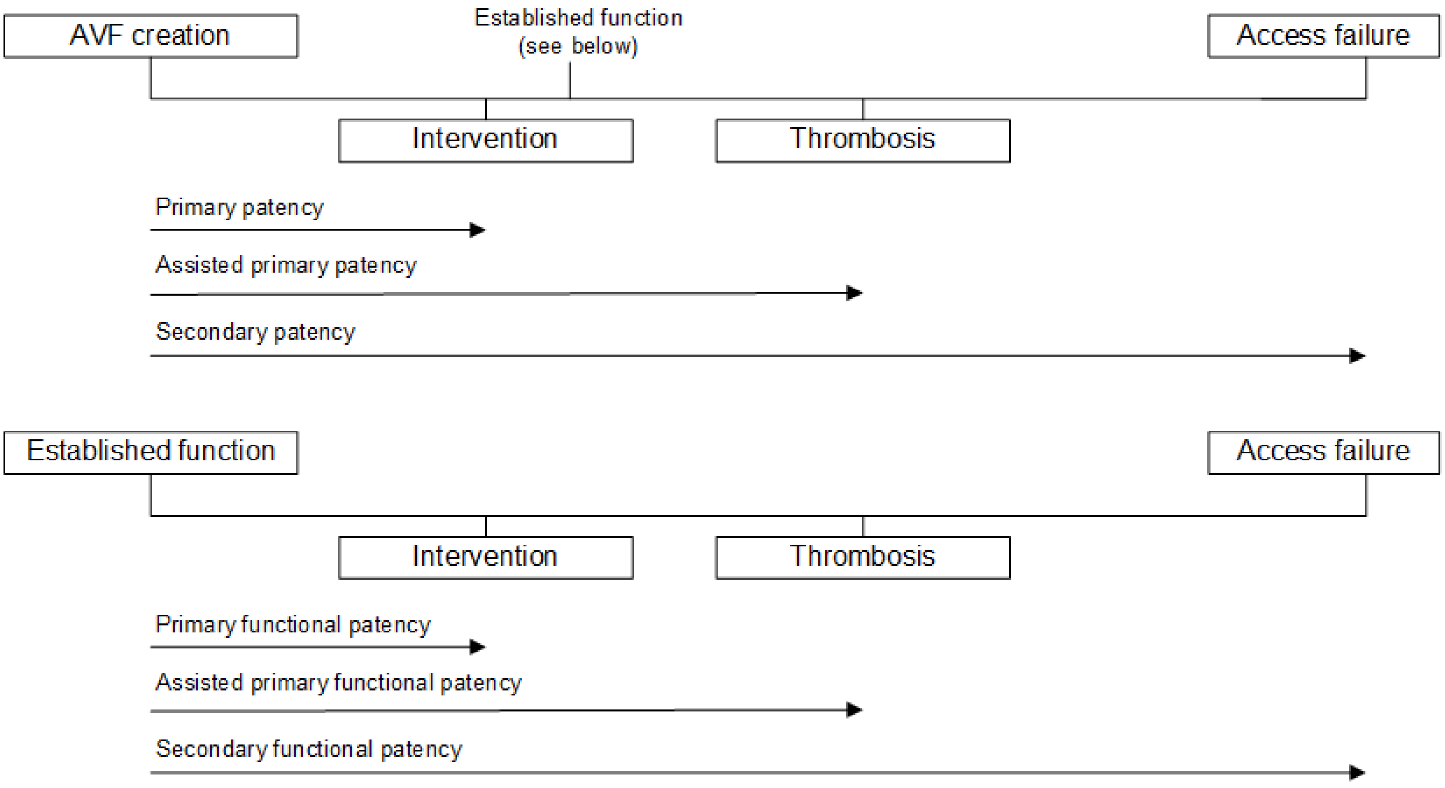

Detailed definitions for established function and patency are found in Supplemental Table 1.

AVF arteriovenous fistula

Supplemental Figure 3: Plot of baseline characteristics

	Proportion (95% CI)			
	Established function		Primary non-function	
**Subgroups**	**N=207**	**P-value**	**N=207**	**P-value**
**Age, y**				
18-44.9	0.67 (0.48, 0.82)	0.35*	0.39 (0.23, 0.58)	0.02*
45-64.9	0.73 (0.62, 0.82)		0.45 (0.34, 0.56)	
65-74.9	0.71 (0.54, 0.84)		0.51 (0.35, 0.67)	
≥ 75	0.60 (0.45, 0.74)		0.63 (0.47, 0.76)	
**Sex**				
Female	0.60 (0.48, 0.71)	<0.05	0.55 (0.43, 0.66)	0.02
Male	0.73 (0.65, 0.81)		0.46 (0.38, 0.55)	
**Dialysis status**				
On HD	0.73 (0.64, 0.81)	0.34	0.43 (0.34, 0.53)	0.13
Non-dialysis CKD	0.64 (0.53, 0.74)		0.57 (0.45, 0.67)	
Other	0.60 (0.26, 0.88)		0.60 (0.26, 0.88)	
**Diabetes**				
No	0.69 (0.59, 0.78)	0.88	0.51 (0.41, 0.62)	0.64
Yes	0.68 (0.59, 0.77)		0.48 (0.38, 0.57)	
**Fistula location**				
Lower arm	0.62 (0.53, 0.71)	0.03	0.59 (0.50, 0.68)	0.001
Upper arm	0.77 (0.67, 0.85)		0.37 (0.27, 0.47)	
**BMI, kg/m^2**				
<18.5	0.71 (0.29, 0.96)	0.64*	0.43 (0.10, 0.82)	0.81*
18.5-24.9	0.69 (0.55, 0.81)		0.47 (0.34, 0.61)	
25-29.9	0.70 (0.58, 0.81)		0.52 (0.40, 0.64)	
≥ 30	0.66 (0.53, 0.77)		0.49 (0.36, 0.61)	
**First permanent access**				
No	0.74 (0.65, 0.81)	0.08	0.42 (0.33, 0.51)	0.02
Yes	0.62 (0.51, 0.72)		0.59 (0.48, 0.69)	

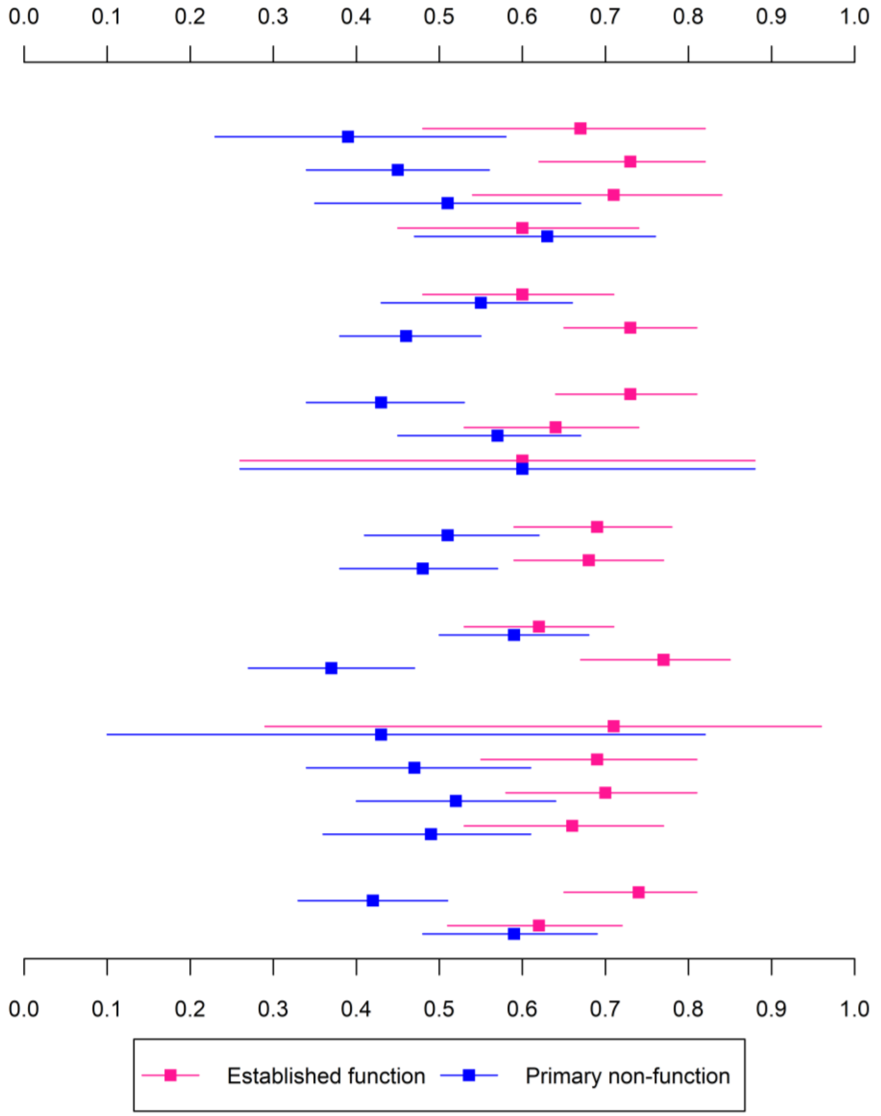

Results presented for 207 of 257 AVF where primary non-function and established function could be evaluated. “On HD” includes participants using CVC or previous AVF/AVG; “Other” includes participants using functioning transplant or PD.

Exact confidence intervals for proportions were computed; P-values based on chi squared tests or trend tests*

AVF arteriovenous fistula; AVG arteriovenous graft; BMI body mass index; CI confidence interval; CKD chronic kidney disease; CVC central venous catheter; HD hemodialysis; PD peritoneal dialysis

Supplemental Table 1: Patency outcomes for AVF that are first permanent access for participant

Outcome	1 year competing risk	1 year Kaplan-Meier	3 year competing risk	3 year Kaplan-Meier	5 year competing risk	5 year Kaplan-Meier
Including only AVF with established function (N=56)						
Loss of primary patency	37.5 (25, 49.9)	38.0 (26.7, 52.2)	67.9 (53.9, 78.4)	76.0 (62.8, 87.2)	67.9 (53.9, 78.4)	76.0 (62.8, 87.2)
Loss of assisted primary patency	3.6 (0.7, 10.9)	3.7 (0.9, 13.9)	19.6 (10.5, 30.9)	23.2 (13.5, 38.2)	23.2 (13.2, 34.9)	29.7 (18.1, 46.5)
Loss of secondary patency	0	0	10.7 (4.4, 20.3)	12.7 (5.9, 26.2)	17.9 (9.2, 28.8)	25.7 (14.3, 43.6)
Including all newly created AVF (N=117)						
Loss of primary patency	44.4 (35.3, 53.2)	48.6 (39.5, 58.6)	64.1 (54.7, 72)	76.9 (67.5, 85.3)	66.7 (57.3, 74.4)	82.7 (72.9, 90.5)
Loss of assisted primary patency	23.1 (15.9, 31)	25.1 (17.9, 34.5)	35.9 (27.3, 44.5)	43.0 (33.6, 53.8)	39.3 (30.5, 48)	51.0 (40.2, 62.8)
Loss of secondary patency	20.5 (13.7, 28.2)	22.2 (15.5, 31.4)	31.6 (23.4, 40.1)	37.4 (28.5, 48.1)	36.8 (28.1, 45.4)	49.2 (38.2, 61.4)

Loss of patency is presented as % (with corresponding 95% CI).

For competing risks analyses, the loss of patency is based on the cumulative incidence. Death, moving out of province, recovery of function, receiving a kidney transplant, starting chronic PD, withdrawal of consent are treated as competing risks. Participants are censored at end of follow-up

For Kaplan-Meier analyses, the loss of patency is based on the 1-KM estimator. Participants are censored for death, moving out of province, recovery of function, receiving a kidney transplant, starting chronic PD, withdrawal consent, or reaching end of follow-up.

Time of patency for the primary, assisted primary and secondary patency measures begins at the time of AVF creation.

AVF arteriovenous fistulas; CI confidence interval; KM Kaplan-Meier; PD peritoneal dialysis

Supplemental Table 2: Other patency outcomes

Outcome	1 year competing risk	1 year Kaplan-Meier	3 year competing risk	3 year Kaplan-Meier	5 year competing risk	5 year Kaplan-Meier
Including only AVF with established function (N=142)						
Loss of primary functional patency	38 (30.1, 45.9)	40.3 (32.5, 49.1)	56.3 (47.8, 64)	66.5 (57.4, 75.4)	59.2 (50.6, 66.7)	73.4 (63.7, 82.3)
Loss of assisted primary functional patency	8.5 (4.6, 13.7)	9.3 (5.4, 15.8)	19 (13, 25.8)	24.2 (17.1, 33.5)	23.2 (16.7, 30.5)	35.6 (25.8, 47.7)
Loss of secondary functional patency	4.9 (2.2, 9.4)	5.5 (2.7, 11.3)	13.4 (8.4, 19.5)	17.7 (11.5, 26.6)	16.2 (10.7, 22.7)	25.2 (16.9, 36.6)

Loss of patency is presented as a % (with corresponding 95% CI).

For competing risks analyses, the loss of patency is based on the cumulative incidence. Death, moving out of province, recovery of function, receiving a kidney transplant, starting chronic PD, withdrawal of consent are treated as competing risks. Participants are censored at end of follow-up

For Kaplan-Meier analyses, the loss of patency is based on the 1-KM estimator. Participants are censored for death, moving out of province, recovery of function, receiving a kidney transplant, starting chronic PD, withdrawal consent, or reaching end of follow-up.

Time of patency for the *functional* patency measures begin at the time of established function. Functional patency measures are only assessed among AVF that matured (i.e., had established function).

AVF arteriovenous fistulas; CI confidence interval; KM Kaplan-Meier; PD peritoneal dialysis

Supplemental Table 3: Reasons for censoring, by outcome

Censoring reason	Primary patency	Assisted primary patency	Secondary patency	Primary functional patency	Assisted primary functional patency	Secondary functional patency
Including only AVF with established function (N=142)						
	**N=45**	**N=101**	**N=110**	**N=55**	**N=101**	**N=110**
Death	26 (57.8)	69 (68.3)	75 (68.2)	31 (56.4)	69 (68.3)	75 (68.2)
Kidney transplant	16 (35.6)	24 (23.8)	26 (23.6)	19 (34.6)	24 (23.8)	26 (23.6)
Final visit for study	2 (4.4)	3 (3.0)	3 (2.7)	2 (3.6)	3 (3.0)	3 (2.7)
Moved	1 (2.2)	4 (4.0)	4 (3.6)	2 (3.6)	4 (4.0)	4 (3.6)
Started chronic PD	0 (0)	1 (1.0)	1 (0.9)	1 (1.8)	1 (1.0)	1 (0.9)
Withdrew consent	0 (0)	0 (0)	0 (0)	0 (0)	0 (0)	0 (0)
Modality switch to conservative care	0 (0)	0 (0)	0 (0)	0 (0)	0 (0)	0 (0)
Recovered function	0 (0)	0 (0)	1 (0.9)	0 (0)	0 (0)	1 (0.9)
Including all newly created AVF (N=257)						
	**N=85**	**N=150**	**N=160**	n/a	n/a	n/a
Death	59 (69.4)	109 (72.7)	115 (71.9)			
Kidney transplant	17 (20.0)	25 (16.7)	27 (16.9)			
Moved	5 (5.9)	8 (5.)	8 (5.0)			
Final visit for study	2 (2.4)	3 (2.0)	3 (1.9)			
Started chronic PD	1 (1.2)	3 (2.0)	3 (1.9)			
Withdrew consent	1 (1.2)	1 (0.7)	2 (1.3)			
Modality switch to conservative care	0 (0)	1 (0.7)	1 (0.6)			
Recovered function	0 (0)	0 (0)	1 (0.6)			

N (%)

Functional patency measures are only assessed among AVF that matured (i.e., had established function).

AVF arteriovenous fistula; n/a not applicable; PD peritoneal dialysis
